# MYCN transcriptionally represses *CD9* to trigger an invasion-metastasis cascade in neuroblastoma

**DOI:** 10.1186/2194-7791-2-S1-A13

**Published:** 2015-07-01

**Authors:** Johannes Fabian, Desirée Opitz, Kristina Althoff, Marco Lodrini, Kathy Astrahantseff, Barbara Hero, Ruth Volland, Anneleen Beckers, Katleen de Preter, Nitin S Patil, Mohammed L Abba, Theresa M Thole, Jasmin Wünschel, Annette Künkele, Jamie Hu, Leonille Schweizer, Gunhild Mechtersheimer, Daniel R Carter, Belamy B Cheung, Odilia Popanda, Andreas von Deimling, Kai-Oliver Henrich, Frank Westermann, Manfred Schwab, Jan Koster, Rogier Versteeg, Glenn M Marshall, Frank Speleman, Margot Zoeller, Heike Allgayer, Matthias Fischer, Frank Berthold, Andreas E Kulozik, Olaf Witt, Angelika Eggert, Johannes H Schulte, Hedwig E Deubzer

**Affiliations:** Clinical Cooperation Unit Pediatric ncology, German Cancer Research Center (DKFZ), INF 280, 69120 Heidelberg, Germany; Department of Pediatric Hematology & Oncology, University Children's Hospital Essen, Hufeland Str. 55, 45147 Essen, Germany; Translational Neurooncology, German Consortium for Translational Cancer Research (DKTK), Partner Site Essen/ Duesseldorf, Essen, Germany; Department of Pediatric Hematology, Oncology & BMT, Charité - University Hospital Berlin, CVK, Augustenburgerplatz 1, 13353 Berlin, Germany; Department of Pediatric Hematology and Oncology, University of Cologne, Kerpener Str. 62, 50937 Cologne, Germany; Center for Medical Genetics Ghent (CMGG), Ghent University Hospital Medical Research Building (MRB), De Pinelaan 185, Ghent, Belgium; Molecular Oncology of Solid Tumors Unit, DKFZ, INF280, 69120 Heidelberg, Germany; Department of Experimental Surgery, Medical Faculty Mannheim, University of Heidelberg, Mannheim, 68135 Germany; Cornell University, 300 Day Hall, Ithaca, NY 14853 USA; Department of Neuropathology, University of Heidelberg, INF 224, 69120 Heidelberg, Germany; Department of Pathology, University of Heidelberg, INF224, 69120 Heidelberg, Germany; Children's Cancer Institute, UNSW, Corner High & Botany Streets, Randwick, New South Wales 2031 Australia; Division of Epigenomics and Cancer Risk Factors, DKFZ, INF 280, 69120 Heidelberg, Germany; Clinical Cooperation Unit Neuropathology, DKFZ, INF280, 69120 Heidelberg, Germany; Neuroblastoma Genetics, DKFZ, INF280, 69120 Heidelberg, Germany; Department of Oncogenomics, Academic Medical Center, University of Amsterdam, Meibergdreef 15, PO Box 22700, 1105 AZ Amsterdam, The Netherlands; Kids Cancer Centre, Sydney Children's Hospital Randwick, High Street, Randwick, New South Wales 2031 Australia; Experimental Surgery and Tumor Cell Biology, University of Heidelberg, INF 365, 69120 Heidelberg, Germany; Center for Molecular Medicine Cologne, University of Cologne, Robert-Koch-Str. 21, 50931 Cologne, Germany; Department of Pediatric Hematology and Oncology, University of Heidelberg, INF 430, 69120 Heidelberg, Germany; Translational Neuro-Oncology, West German Cancer Center (WTZ), University Hospital Essen, University Duisburg-Essen, Hufeland Str. 55, 45147 Essen, Germany; Center for Medical Biotechnology, University Duisburg-Essen, 45117 Essen, Germany

## Meeting abstract

The systemic and resistant nature of neuroblastoma metastasized to distant organs makes it largely incurable with current multimodal treatment. Clinical progression stems mainly from an increasing burden of metastatic colonization. Novel therapeutic perspectives may be won by blocking as yet poorly understood pathways triggering the migration-invasion-metastasis cascade in neuroblastoma. The CD9 cell surface glycoprotein was decoded as a major downstream player and direct target of the recently described *GRHL1* tumor suppressor in in-depth transcriptome analyses and ChIP-qRT-PCR. CD9 is known to facilitate carcinoma cell motility and metastasis. High-level CD9 expression in primary neuroblastomas correlated with patient survival and established markers for favorable disease. Low-level *CD9* expression was an independent risk factor for adverse outcome and predicted poor treatment response in patients with the worst outcome. MYCN and HDAC5 co-localized to the *CD9* promoter and repressed transcription. *CD9* expression was strongly reduced during progressive development of murine tumors in the *TH-MYCN* transgenic mouse model of neuroblastoma compared to expression in ganglia from wildtype mice, further supporting MYCN involvement in *CD9* transcriptional repression in neuroblastoma cells. We detected differential *CD9* methylation in 450K methylation array analyses of primary neuroblastomas, and *CD9* hypermethylation was associated with reduced *CD9* expression, supporting epigenetic regulation. Inducing CD9 expression in a SH-EP cell model inhibited migration and invasion in Boyden chamber assays. Enforced CD9 expression in neuroblastoma cells transplanted onto chicken chorioallantoic membranes strongly reduced metastasis to chicken embryo bone marrow. Combined treatment of neuroblastoma cells with inhibitors for HDACs and DNA methyltransferase induced CD9 expression. Our results show CD9 is a critical and indirectly druggable mediator of neuroblastoma cell invasion and metastasis.

